# Low-Complexity Beamforming Design for a Cooperative Reconfigurable Intelligent Surface-Aided Cell-Free Network

**DOI:** 10.3390/s23020903

**Published:** 2023-01-12

**Authors:** Muhammad Zain Siddiqi, Aisha Munir, Syed Agha Hassnain Mohsan, Shashi Shah, Sushank Chaudhary, Paramin Sangwongngam, Lunchakorn Wuttisittikulkij

**Affiliations:** 1 Wireless Communication Ecosystem Research Unit, Department of Electrical Engineering, Chulalongkorn University, Bangkok 10330, Thailand; 2 Optical Communications Laboratory, Ocean College, Zhejiang University, Zhoushan 316021, China; 3 Spectroscopic and Sensing Devices Research Group, National Electronics and Computer Technology Center, Bangkok 12120, Thailand

**Keywords:** cell-free, reconfigurable intelligent surface, cooperative communication, low-complexity, beamforming, particle swarm optimization, metasurface

## Abstract

Cell-free (CF) networks are proposed to suppress the interference among collocated cells by deploying several BSs without cell boundaries. Nevertheless, as installing several base stations (BSs) may require high power consumption, cooperative CF networks integrated with a reconfigurable intelligent surface (RIS)/metasurface can avoid this problem. In such cooperative RIS-aided MIMO networks, efficient beamforming schemes are essential to boost their spectral and energy efficiency. However, most of the existing available beamforming schemes to maximize spectral and energy efficiency are complex and entail high complexity due to the matrix inversions. To this end, in this work we present a computationally efficient stochastic optimization-based particle swarm optimization (PSO) algorithm to amplify the spectral efficiency of the cooperative RIS-aided CF MIMO system. In the proposed PSO algorithm, several swarms are generated, while the direction of each swarm is tuned in each iteration based on the sum-rate performance to obtain the best solution. Our simulation results show that our proposed scheme can approximate the performance of the existing solutions for both the performance metrics, i.e., spectral and energy efficiency, at a very low complexity.

## 1. Introduction

The existing wireless communication setup is based on cellular architecture, where several base stations (BSs) are deployed to cover an area. The transmission coverage of the BSs is usually limited to a hexagonal cell, and cannot be extended due to power constraints applied at the BSs. However, such a cellular network architecture causes severe inter-cell interference that results in serious performance loss in network capacity [1]. This is mainly because the users located at the edge of the cell boundaries experience strong interference, thus limiting the capacity of the cellular network [2]. To avoid this issue, a coordinated/cooperative beamforming approach for the BSs approach was adopted in [3]. In cooperative communication, several BSs jointly transmit signals in the downlink direction to serve multiple users. The network densification at a large scale ensures a better spectral efficiency compared to the single BS scenario. However, these BSs still operate in their respective cells to serve multiple users and yield unavoidable interference in the adjacent cells.

Recently, cell-free (CF) networks were proposed to circumvent the problem of inter-cell interference [4]. In a CF network, a large number of BSs, employed with a single antenna or multiple antennas, are deployed over an area to serve multiple users. These BSs jointly transmit the signals coherently to serve the users without cell boundaries, thus significantly improving spectral efficiency [5]. To further improve the performance of CF networks, various solutions such as beamforming [6], power allocation [7], and channel estimation [8] have been proposed. Nevertheless, deploying a large number of BSs to cover a certain area requires a significant cost and power consumption that restricts the cellular vendors to deploy several BSs at a large scale.

Recently, a low-cost spectral- and energy-efficient reconfigurable intelligent surface (RIS) technology was proposed [9,10,11]. Different from the existing wireless technologies that work on adapting the radio channels as per the radio condition, RIS has the potential to control the radio environment through its phase-shifting elements [12]. The RIS phase-shifting elements are composed of special metamaterial elements that have the intrinsic property to control the propagation environment. Benefiting from the low power consumption of the RIS elements, this method is attracting attention from academia and industry in various use cases, such as wireless power transfer (WPT) [13], physical layer security (PLS) [14,15,16], and cognitive radio networks [17]. Additionally, at higher frequencies (e.g. millimeter/terahertz wave bands), the higher path loss (PL) severely impacts the performance of the users; thus, RIS can be deployed to improve the QoS of the users that suffer from the unavoidable PL problem [18,19]. Moreover, RIS is also being used with unmanned aerial vehicles (UAVs) in remote areas such as hill stations to maintain long-distance communications [20]. To make wireless systems more energy efficient, RIS can be combined with backscatter communication [21]. Usually, RIS does not comply with the complex radio frequency (RF) chains; hence, no noise or thermal heat is generated at the RIS. Thus, these phase-shifting elements only redirect the incident beam/s toward the user(s) direction, realizing high beamforming gain with reconfigurable parameters. Taking advantage of this working principle, we can selectively replace some BSs with the RISs to maximize the spectral efficiency of the cooperative wireless network, while realizing the objective of green communications.

### 1.1. Related Works

To date, several works have been reported that consider RIS as a substitute for the amplifying and forward (AF) relay to achieve a high-performance gain in the spectral efficiency of future wireless networks. Specifically, the authors in [22] proposed a semidefinite relaxation (SDR) method to boost the spectral efficiency of the RIS-aided MIMO wireless systems. The SDR approach is effective and ensures a considerable performance gain but at the cost of high computational complexity. A fractional programming (FP) method was proposed in [23] to maximize the weighted sum rate of the RIS-aided network. Moreover, the authors of [24] proposed the sequential fractional programming (SFP) method to enhance the spectral/energy efficiency of the RIS-aided MIMO scenario. To realize this objective, they optimize the passive beamforming matrix at the RIS and the active beamforming matrix at the BS to achieve the high spectral efficiency performance of the RIS-aided system. Moreover, the authors of [25] proposed a block coordinate descent (BCD) method to alternatively optimize the beamforming matrices to maximize the weighted sum rate of the wireless system. In the continuation of the same objective, the authors of [26] presented a penalty dual decomposition (PDD) method to optimize the passive beamforming of the RIS-aided MIMO system. They proposed a two-time scale (TTS) beamforming method to maximize the sum rate of the RIS-aided system under the Rician channel model. Although all of the aforementioned solutions ensure a considerable spectral efficiency performance, the complexity of these solutions is too high, which restricts them to be considered in their practical hardware realization. Thus, an alternate computationally efficient algorithm is required which could easily be realizable in the practical hardware in future 6G communications. We have summarized the existing algorithms with their pros and cons in Table 1.

### 1.2. Contribution

Motivated by the previous discussion, in this work we present the low-complexity beamforming designs at the BSs and the RISs for a cooperative RIS-aided communication system to maximize the network capacity. It is worth mentioning here that the same low-complexity algorithm can be extended to another performance indicator (energy efficiency) to realize the objective of green transmission.

- At first, we formulate the network capacity as well as energy efficiency maximization problem for the cooperative RIS-aided CF MIMO framework under the limitations of power as well as per element hardware constraints.
- We propose a computationally efficient iterative stochastic optimization-based particle swarm optimization (PSO) method to solve the capacity maximization problem. We adopt PSO to optimize the passive beamformer at the RISs and apply a nulling algorithm at the BSs to realize the objective of the proposed problem. Specifically, the PSO algorithm is based on the number of possible solutions, and these solutions are then optimized to obtain a better solution among all possible solutions.
- In the end, the proposed solution is evaluated using several numerical computations, and the results indicate that the performance of the proposed solution is almost the same as that of the existing solution for both scenarios (spectral and energy efficiency) but at significantly low complexity.

### 1.3. Organization and Notations

*Organization*: The rest of the paper is organized as follows. The system model of the cooperative RIS-aided CF network followed by the problem formulation is discussed in Section 2. Section 3 contains the joint beamforming solution to solve the network optimization problem. To support the hypothesis, the simulation results are discussed in Section 4. In the end, Section 5 contains the conclusion.

*Notations*: All the main notations of the paper are summarized in Table 2.

## 2. System Model and Problem Formulation

At first, we illustrate the system model for cooperative communications, and then we formulate the network optimization problem for the RIS-aided CF MIMO network.

### 2.1. System Model

**Figure 1:**
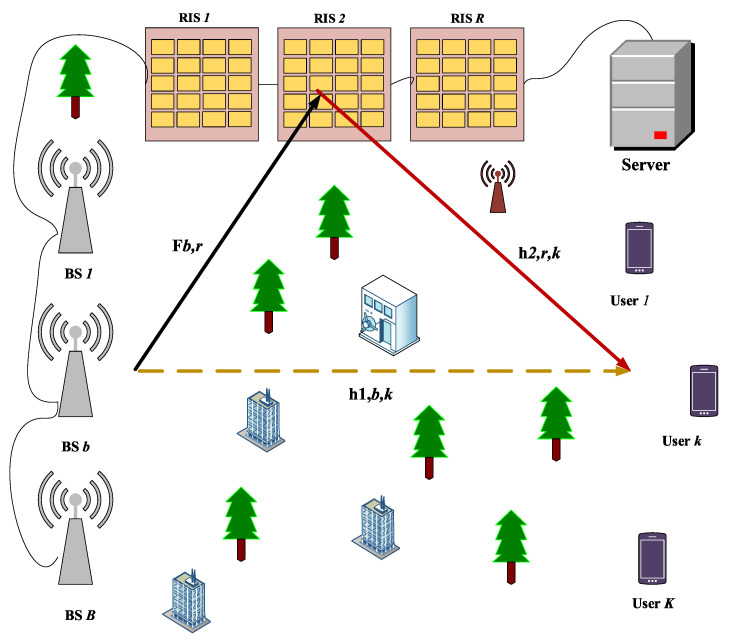
A RIS-aided cooperative CF network.

We consider a downlink transmission model, where a number of BSs along with several RISs jointly transmit the radio signals to the K users. These BSs and RISs are deployed using a cell-free (CF) approach, where BSs and RISs cooperate to serve multiple users without cell boundaries, eliminating the problem of cell-edge users. This means that each user will receive a good quality signal either from the BSs or RISs or even receives a combined signal from both BSs and RISs, which improves the performance of the RIS-aided wireless systems. It is worth mentioning here that whenever a user experiences a large-scale fading problem due to the presence of high-rise buildings in the urban area or due to the green belts that come between the BSs and the users, a good LoS link between the BSs and users may be affected, which degrades the performance of the wireless system. Therefore, we introduce an RIS, which is installed at high-rise buildings between the BS and a user to construct an extra link between the BSs and the users. Thus, an indirect path will always be available even if the direct link will be under a deep fade. Additionally, these BSs and RISs are connected to the central processing unit (CPU) via wireless/fiber links. A CPU is used to control the BSs and RISs operations remotely by instructing different commands. Considering M number of antenna elements at the BSs and N phase-shifting elements at the RISs, the overall system model is illustrated in Figure 1.

### 2.2. Transmitter

In the cooperative environment of the CF system model, all the BSs and RISs are synchronized to produce a coherent transmission to serve *K* users. Hence, here we assume the fact that all the BSs jointly transmit the symbol intended to the *k*th user. Let the *b*th BS transmit the *k*th symbol, $s_{k}$, with a normalized unit power gain to the *k*th user, where *k* ∈1,2,…,K. Then, for all *K* users, the transmitted signal **x** $\in C^{Mx1}$ from the *b*th BS is represented by

(1) $$\begin{array}{c} x_{b}=\sum_{k=1}^{K}g_{b,k}s_{k}, \end{array}$$

where $g_{b,k}\in C^{Mx1}$ is the beamforming from the *b*th BS for the *k*th user. In general, the transmitted power of the BS is limited; hence, the signal transmitted from the *b*th BS will have a power limitation, which is defined as

(2) $$E\left(\|x_{b}{\|}_{2}^{2}\right)=\mathrm{Tr}\left(G_{b}^{H}G_{b}\right)\leq P_{b},$$

where $G_{b}\in C^{MxK}$ denotes the active beamforming, ∀K, from the *b*th BS; $P_{b}$ represents the power budget for the *b*th BS. Since we exploit *B* BSs in our framework, we use the same power budgets for all *B* BSs.

### 2.3. Receiver

The received signal $y_{b,k}$ from the *b*th BS at the *k*th user is given by

(3) $$\begin{array}{c} \begin{array}{cc} \; & y_{b,k}=h_{1,b,k}x_{b}+\sqrt{\alpha_{k}}\sum_{r=1}^{R}h_{2,r,k}\Psi_{r}F_{b,r}x_{b}+n_{k},\\ & =\left(h_{1,b,k}+\sqrt{\alpha_{k}}\sum_{r=1}^{R}h_{2,r,k}\Psi_{r}F_{b,r}\right)\sum_{j=1}^{K}g_{b,k}s_{j}+n_{k}, \end{array} \end{array}$$

where $h_{1,b,k}\in C^{1xM}$ denotes the direct channel link between the *b*th BS to the *k*th user, and α denotes the channel attenuation coefficient. The $h_{2,r,k}\in C^{1xN}$ is the second channel link, which is between the *r*th RIS and the *k*th single antenna user. The collocated LoS channel between a BS and an RIS is designated with $F_{b,r}\in C^{NxM}$. This link ensures an extra degree of freedom for the users to enjoy the services even if the direct link between the BSs and the users is not available. We comprehensively discuss the channel models for both direct and indirect links in the simulation section. Moreover, the beamforming matrix $\Psi_{r}$ of the *r*th RIS is a diagonal matrix and can be defined as $\Psi_{r}=\mathrm{diagonal}\left(\Psi_{r,1},\Psi_{r,2},\ldots ,\Psi_{r,N}\right)$, where the *n*th RIS element $\Psi_{r,n}$ satisfies the unit modulus constraint ∀n∈{1,2,…,N}. The final term $n_{k}$ denotes the additive white Gaussian noise (AWGN) at the *k*-th user.

Based on (3), considering all *B* BSs, the signal-to-interference-plus-noise ratio (SINR) for the *k*-th user can be written as

(4) $$\begin{array}{c} {\mathrm{SIN}R}_{k}=\frac{\sum_{b=1}^{B}{\left|h_{1,b,k}+\left(\sqrt{\alpha_{k}}\sum_{r=1}^{R}h_{2,r,k}\Psi_{r}F_{b,r}\right)g_{b,k}\right|}^{2}}{\sum_{b=1}^{B}\sum_{j=1,j\neq k}^{K}{\left|h_{1,b,k}+\left(\sqrt{\alpha_{k}}\sum_{r=1}^{R}h_{2,r,k}\Psi_{r}F_{b,r}\right)g_{b,j}\right|}^{2}+\sigma^{2}}. \end{array}$$

Finally, ∀*K* users, the sum rate $R_{tot}$ in the RIS-aided cooperative CF network can be defined as

(5) $$R_{tot}=\sum_{k=1}^{K}\log_{2}\left(1+{\mathrm{SIN}R}_{k}\right).$$

### 2.4. Problem Formulation

In this subsection, we formulate the network capacity maximization problem for the cooperative RIS-aided communication network such that the power limit condition at the *b*th BS and the per-element modulus constraint at the *r*th RIS could be fulfilled. To achieve this goal, we jointly design the beamforming matrices $G_{b}$ and $\Psi_{r}$ at the *b*th BS and the *r*th RIS, respectively. Mathematically, the total sum-rate optimization problem of the cooperative network is then formulated as

(6) $$\begin{array}{c} \max_{\Psi_{r},G_{b}}\;R_{tot}\\ \;\;\;\;\;s.t.\Psi \in F,\\ \;\;\;\;\;\;\;\;\;\;\mathrm{Tr}\left(\left(G_{b}\right){\left(G_{b}\right)}^{H}\right)\leq P_{b}, \end{array}$$

where F denotes the codebook that contains all possible passive beamformers satisfying per element modulus constraint. It is important to mention here that in this work, we consider the scenario where the BS knows the channel state information (CSI) of both direct and indirect channel links, and the CSI can be obtained from either technique discussed in [27]. Moreover, after acquiring the CSI, all the BSs implement ZF, also called a nulling algorithm, to nullify the inter-user interference while transmitting the signals to the intended *k*th user. Additionally, since we use less number of antennas at the *B* BSs; however, employing a large number of phase-shifting elements over the RIS surface helps us in eliminating the interference among the *K* users at the BSs.

For the sake of convenience, defining Ψ = $\mathrm{diag}(\Psi_{1},\Psi_{2},\ldots ,\Psi_{R})$, $H_{2,R}=$${\left[\sqrt{\alpha_{1}}h_{2,1,1}^{T},\sqrt{\alpha_{2}}h_{2,2,2}^{T},\ldots ,\sqrt{\alpha_{K}}h_{2,R,K}^{T}\right]}^{T}$, $F_{b}={\left[F_{b,1},F_{b,2},\ldots ,F_{b,R}\right]}^{T}$, and finally, $H_{1,b}={\left[h_{1,b,1}^{T},h_{1,b,2}^{T},\ldots ,h_{1,b,K}^{T}\right]}^{T}$. Then, the equivalent channel $W_{b}=H_{1,b}+\sqrt{\alpha_{1}}H_{2,r}\Psi F_{b}$. Then, by applying pseudo inverse on the equivalent channel matrix $W_{b}$, we can obtain the beamforming matrix of the *b*th BS $G_{b}$, i.e., $G_{b}=W_{b}^{†}$. Replacing the $G_{b}=W_{b}^{†}$ into (6), we obtain the following sum rate $R_{tot}$ optimization problem

(7) $$\begin{array}{c} \max_{\Psi ,\beta }\;R_{tot}\\ \;\;\;\;\;s.t.\Psi_{n}=\left|1\right|,\\ \;\;\;\;\;\;\;\;\;\;\mathrm{Tr}\left({\left({\bar{W}}_{b}\right)}^{†}{\left(\bar{W}\right)}_{b}^{†H}\right)\leq \beta P_{T}, \end{array}$$

where β denotes the power scaling factor. Hence, with this, the optimal beamforming at the BSs can be obtained as ${\hat{G}}_{b}=\sqrt{\frac{1}{\beta }}G_{b}$. Nonetheless, the per unit modulus constraint for all *R* RISs in the reformulated problem (7) is still making the problem a non-convex problem, i.e., $\Psi_{n}=$$\left|1\right|$, where n=1,2,…,RN. Thus, to meet the hardware unit modulus constraint, for all *R* RISs we propose a low-complexity iterative optimization scheme.

## 3. Passive Beamforming Design

In this section, we present an iterative low-complexity stochastic optimization particle swarm optimization (PSO) algorithm, which is designed based on the social behavior of the birds that fly in a cluster to find their best target to feed themselves [28]. Inspired by this key idea, in our sum-rate optimization problem, we employ the PSO algorithm to optimize the active and passive beamforming matrices of the BSs and RISs, respectively.

### 3.1. PSO-Based Passive (RIS) Beamformer

The PSO is a heuristic algorithm that works on predefined solutions. These solutions are iteratively refined to yield a suboptimal solution. This technique became popular due to its simple implementation and fast convergence toward the desired solution. The PSO algorithm can trap a local-optimal solution in a large dimensional space and yield a near-optimal solution in a reasonable time; thus, PSO can easily be realized in a practical system. Due to its simple working principle, it is widely applied in various engineering disciplines to solve engineering problems.

Specifically, in the PSO algorithm, a number of swarms/populations called particles are initialized. Usually, these particles are initialized within the cardinality of swarms/population size based on some initial velocities. At any instant in time, the coordinate of a particle represents the position of a particle. These positions of the particles are used to obtain the objective function value (OBV) of the problem. In each iteration, whenever a new position is attained based on the updated velocity by the swarms, a new OBV is attained. This velocity is refined based on the best position of the particles to obtain a better result by the end of the iterations.

The entire PSO algorithm to solve problem (7) is summarized in Algorithm 1. Specifically, the PSO algorithm works on the following steps. To begin with, we generate *S* swarms at random positions as ${\bar{\Psi }}_{1}\left(0\right),{\bar{\Psi }}_{2}\left(0\right),\ldots ,{\bar{\Psi }}_{S}\left(0\right)$ in the first iteration. These swarms are called passive beamformers in our problem. To ensure unity power, the *S* positions are normalized. Then, the velocity of all *S* swarms is randomly generated as $v_{1}\left(0\right),v_{2}\left(0\right),\ldots ,v_{S}\left(0\right)$, where we denote the velocity of the *s*th swarm with $v_{s}$. For each *s*th passive beamformer ${\bar{\Psi }}_{s}$ we compute the active beamforming matrix $G_{b}$, which is obtained from the ${\bar{W}}_{b}$ for the *b*th BS. ∀S swarms, compute the OBV, i.e., sum rate $R_{tot}$ (7) in our problem. We compare the OBV ∀S, and find the $s^{*}$th position passive beaformer ${\bar{\Psi }}_{s^{*}}$ that maximizes the OBV in (7). This highest OBV container passive beamformer ${\bar{\Psi }}_{s^{*}}$ is denoted with ${\bar{w}}_{\mathrm{best}}$, and is known as the best particle position (passive beamformer) in one iteration. In the subsequent iteration, we refine the velocity of the *s*th swarm, and then upon this new velocity we refine the *s*th swarm position (${\bar{\Psi }}_{s}$ passive beamformer in our case) as

(8) $$\begin{array}{c} v_{s}\left(i+1\right)=v_{s}\left(i\right)+c_{1}u_{1}⊙\left({\bar{\Psi }}_{s_{\mathrm{best}}}\left(i\right)-{\bar{\Psi }}_{s_{\mathrm{current}}}\left(i\right)\right)\\ +c_{2}u_{2}⊙\left({\bar{w}}_{\mathrm{best}}-{\bar{\Psi }}_{s_{\mathrm{current}}}\left(i\right)\right), \end{array}$$

(9) $${\bar{\Psi }}_{s}\left(i+1\right)={\bar{\Psi }}_{s}\left(i\right)+v_{s}\left(i+1\right),$$

where $c_{1}$ and $c_{2}$ are the positive acceleration coefficients that have the tendency to move the *s*th swarm towards the best position. $u_{1}$ and $u_{2}$ are the uniformly distributed random vectors, while ${\bar{\Psi }}_{s_{\mathrm{best}}}$ and ${\bar{\Psi }}_{s_{\mathrm{current}}}$ are the local best and current best particle position, respectively. Finally, ⊙ represents the element-wise multiplication of the vectors. In the beginning, when *i* = 0, $\forall S{\bar{\Psi }}_{s_{\mathrm{best}}}$(0) = ${\bar{\Psi }}_{s_{\mathrm{current}}}$(0). While in the subsequent iterations, each swarm maintains a record of its own best position, which depends on achieving the highest OBV in (7). Whenever a *s*th passive beamformer updates its position based on updated velocity, its OBV is updated accordingly. If the new OBV (which is based on the new passive beamformer position) is greater than the previous best OBV, then ${\bar{\Psi }}_{s_{\mathrm{best}}}\left(i\right)$ = ${\bar{\Psi }}_{s_{\mathrm{current}}}\left(i\right)$. Finally, ∀S we compare OBV values, and the passive beamformer that has the largest OBV (7), i.e., the best beamformer ${\bar{\Psi }}_{s^{*}}$, will take place the position of ${\bar{w}}_{\mathrm{best}}$, i.e., ${\bar{w}}_{\mathrm{best}}$ =${\bar{\Psi }}_{s^{*}}$. The algorithm continues until all the iterations *I* are satisfied. Then, finally, the obtained beamformer is normalized as Ψ = $\frac{{\bar{\Psi }}_{s_{\mathrm{best}}}}{\sqrt{{\bar{\Psi }}_{s_{\mathrm{best}}}^{H}{\bar{\Psi }}_{s_{\mathrm{best}}}}}$. It is worth mentioning here that the obtained passive beamformer Ψ might not follow the unit modulus constraint; therefore, to ensure the per unit modulus constraint, we set the passive beamformer as $\Psi =e^{j∠\Psi }$. We repeat this process for all *B* BSs to acquire the passive beamformers for *R* RISs.
**Algorithm 1** Passive beamforming design based on PSO.**Input:** Channel matrices $H_{1,b}$, $H_{2,r}$, $F_{b}$; Total no. of iterations *I*; Swarms/Particles size *S*; Number of *B* BSs; Number of *R* RISs; **Output:**
Ψ *Phase 1:*
  1:Randomly initialize *S* swarms as ${\bar{\Psi }}_{1}\left(0\right),{\bar{\Psi }}_{2}\left(0\right),\ldots ,{\bar{\Psi }}_{S}\left(0\right)$ at initial velocities $v_{1}\left(0\right),v_{2}\left(0\right),\ldots ,v_{S}\left(0\right)$;  2:∀*S*, calculate active beamforming matrices $G_{b}$ from equivalent channel matrix $W_{b}$ = $H_{1,b}$+ $\sqrt{\alpha }$$H_{2,r}\bar{\Psi }F_{b}$ for the *b*th BS;  3:∀*S*, compute the objective function value (OBV), i.e., sum-rate $R_{tot}$ to maximize (7);  4:Find ${\bar{w}}_{\mathrm{best}}$ from the sample space of *S*;*Phase 2 (Iterations):*
  5:**for***s* = 1:*S* **do**  6:   Update velocity of *s*th swarm using (8);  7:   Based on updated velocity from Step 6, refine the *s*th swarm position via (9);  8:   Refine OBV value based on this new position;  9:   if (updated OBV > previous best OBV value);   ${\bar{\Psi }}_{s_{\mathrm{best}}}\left(i\right)$ = ${\bar{\Psi }}_{s_{\mathrm{current}}}\left(i\right)$;10:**end for**11:Find ${\bar{w}}_{\mathrm{best}}$;12:i←i+1


### 3.2. Computational Complexity

In this subsection, we present the computational complexity of the proposed PSO algorithm and compare its complexity with the existing scheme [23]. It can be seen that the major contribution to the complexity of the PSO algorithm is on the computations of OBV of *S* passive beamformers (particles) for *K* users. Hence, the overall complexity of the algorithm is O(SKlog(RN)), whereas the complexity entailed by the existing scheme in [23] is $O(R^{3}N^{3}+R^{2}N^{2}+RN)$. This indicates that the complexity entailed by the scheme proposed in [23] grows significantly when the *N* grows to a larger value for a fixed *R*. On the other hand, the complexity of the PSO algorithm increases linearly with the swarm numbers *S* for a fixed *R*. Thus, this gives a major benefit of using the algorithm to realize it in future wireless communication systems.

## 4. Simulation Results and Channel Model

In this section, we present simulated results of the considered framework under different simulation setups to validate the performance of the cooperative RIS-aided CF network.

**Figure 2:**
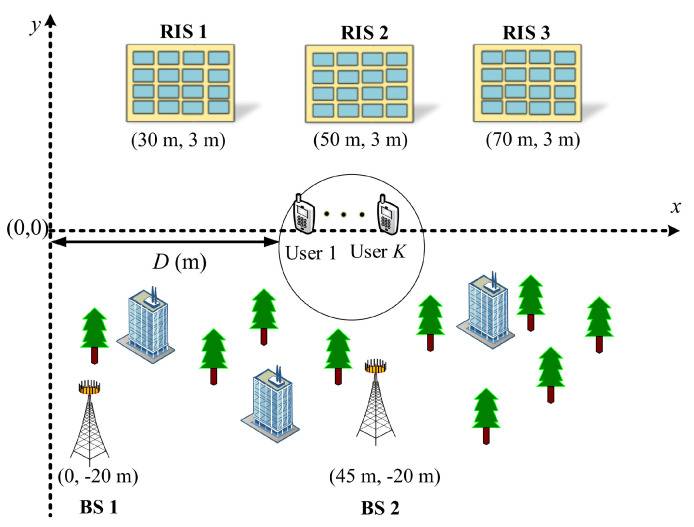
A 2D modeling of the cooperative RIS-aided communication framework.

**Figure 3:**
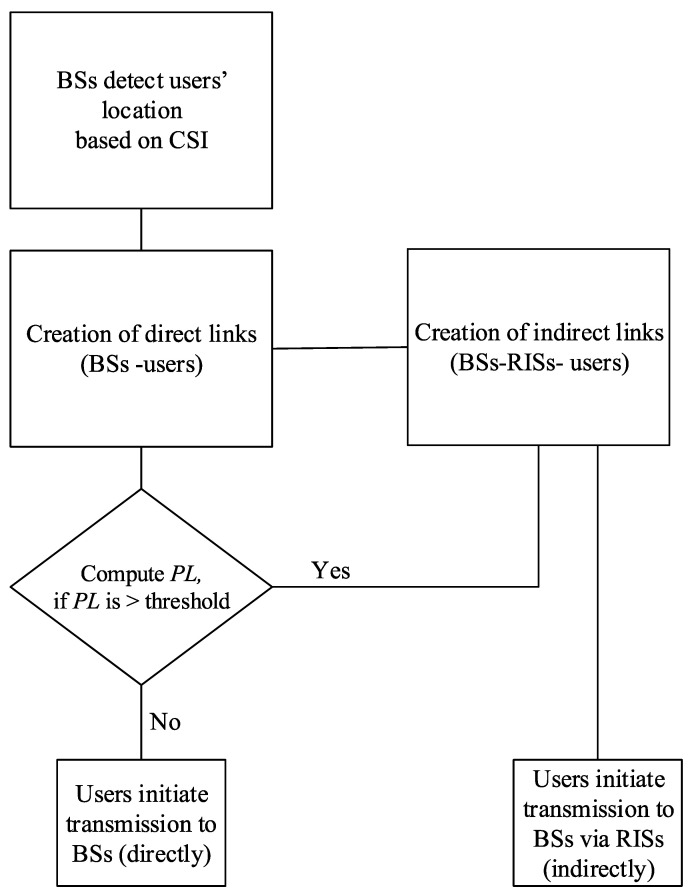
Flow chart diagram of the working principal of the 2D framework.

To further give an insight into the considered framework, we simulate the 2D modeling of the considered cooperative RIS-aided system, which is illustrated in Figure 2. The major benefit of using 2D modeling is that it helps us conceptually visualize the considered cooperative RIS-aided framework. The 2D model of the considered system model consists of two BSs, three RISs, and four single-antenna users. Generally, the users are mobile, and the channel link quality between the BSs and the users can be limited due to the presence of high-rise buildings and green belts, thus creating a serious impact on the performance of the wireless networks. To tackle this situation, we install three RISs onto the three separate buildings to create a degree of freedom to construct an LoS link between RISs and users, which means that users can communicate with the BSs via RISs. We consider that the case where the *b*th BS is located at the distance of (45(b−1) m, −20 m) and the *r*th RIS is distanced at the location of (20 + 10r m, 3 m), where *r* $\in \left(1,3,5\right)$. Moreover, the number of users is randomly and uniformly distributed within a radius of 1 m, and this uniform region is represented by a horizontal distance *D*. To give further insight into 2D modeling, we draw a flow chart diagram to show the entire working process of user association with the BSs and RISs in Figure 3.

### 4.1. Simulation Configurations

In our simulations, we set the antenna elements *M* for all *B* BSs as *M* = 4, while the phase-shifting elements *N* of *R* RISs are set to 48. The number of users *K* is set to *K* = 4, and the cardinality of particle swarms *S* is set as 40. We set the number of iterations *I* = 100, the attenuation coefficient α is set to α = 1, ∀K, and finally, the noise is set to −120 dBm.

### 4.2. Channel Model

To realize the PL in our simulations, we exploit the large-scale channel model which is explicitly discussed in [23,24,29]. ${\hat{d}}_{BS,k}$ represents the distance between the BS and the *k*th user, while we denote the BS to RIS distance as ${\hat{d}}_{BS,RIS}$, and finally, we indicate the distance between the *r*th RIS and the *k*th user with ${\hat{d}}_{RIS,k}$. With these parameters settings, the distance-dependent PL can then be defined as

(10) $$\begin{array}{c} PL\left(\bar{d}\right)=L_{0}{\left(\frac{\bar{d}}{d_{0}}\right)}^{x},\bar{d}\in \left({\hat{d}}_{BS,k},{\hat{d}}_{BS,RIS},{\hat{d}}_{RIS,k}\right) \end{array}$$

where $L_{0}$ is the PL at $d_{0}$ = 1 and its value depends on the wavelength, channel quality, antenna gain, and effective aperture of the antenna [24]. *x* denotes the PL exponent, and the PL exponent between the BS-user is 3, we set the same exponent between the BS-RIS and RIS-user links as 2.0. Additionally, to incorporate small-scale fading, we exploit the Rician fading channel model. Then, the collocated LoS channel between BS and RIS is modeled as [23]

(11) $$F_{BS,RIS}=\sqrt{\frac{\gamma_{\mathrm{BS},RIS}}{1+\gamma_{\mathrm{BS},RIS}}}F_{BS,RIS}^{\mathrm{LoS}}+\sqrt{\frac{1}{1+\gamma_{\mathrm{BS},RIS}}}F_{BS,RIS}^{\mathrm{NLoS}},$$

where $\gamma_{BS,RIS}$ denotes the Rician factor, while **F**${}^{\mathrm{LoS}}$ and **F**${}^{\mathrm{NLoS}}$ represent the LoS and NLoS fading components, respectively. Using the same model, we can generate the small-scale fading channels with corresponding Rician factors for the BS-user and RIS-user links. The simulation parameters are summarized in Table 3.

### 4.3. Spectral Efficiency Performance of Cooperative Network

**Figure 4:**
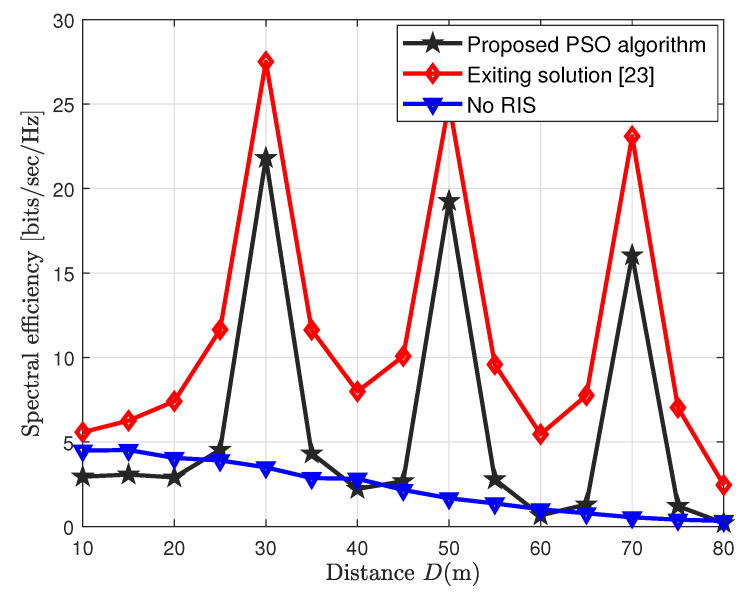
Spectral efficiency vs. wide ranges of *D*.

Figure 4 shows an interesting observation regarding the spectral efficiency performance of the cooperative CF network, which is plotted on the different *D* ranges. We observe from Figure 4 that deploying the RISs between the users and the BSs, such that a cooperative enhancement of the transmission could be established, generates fine peaks in spectral efficiency wherever the RISs are located. Thus, we observe three peaks at different RIS locations, i.e., *D* = 30 m, 50 m, and 70 m. These peaks show the spectral efficiency performance of the proposed low-complexity PSO algorithm and the existing solution [23] tends to reach high spectral efficiency peaks whenever the users pass by the nearest RIS. These RISs, in fact, create an extra link between the users and BSs to improve the spectral efficiency of the RIS-aided CF MIMO network.

### 4.4. Impact of Spectral Efficiency Performance on Different Transmit Power Ranges

**Figure 5:**
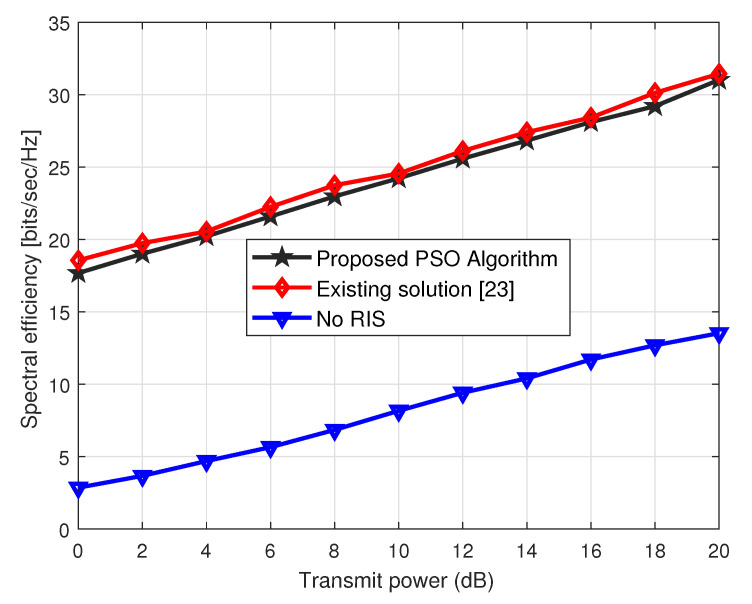
Spectral efficiency performance vs. transmit power (dBs) for *N* = 32.

The spectral efficiency performance of the RIS-aided cooperative CF network is indicated in Figure 5, where we plot the spectral efficiency on the different ranges of the transmit power at the BSs. Here, we set the phase-shifting elements at the *r*th RIS is *N* = 32. From Figure 5, we observe that both the schemes, the proposed and existing solution [23], show the same trend over the entire ranges of the transmit power at the BSs. Moreover, the performance gain achieved by the proposed low-complexity solution is around 94% of the existing scheme [23]. This shows that our proposed scheme tends to approximate the same spectral efficiency performance as achieved by the [23] at a very low complexity. On the other hand, the blue curve represents the case when there is no RIS in the CF network. We can see that performance of the curve always remains low over the entire range of the transmit power at the BS.

**Figure 6:**
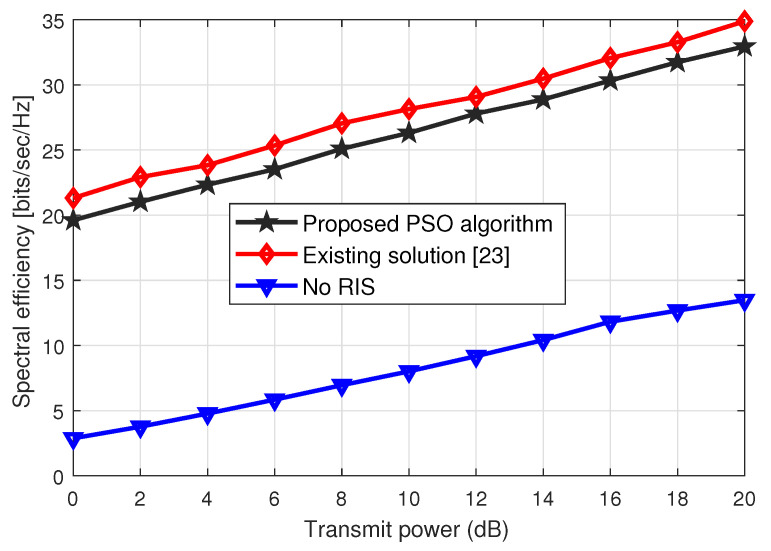
Spectral efficiency performance vs. transmit power (dBs) for *N* = 48.

Next, we plot the same spectral efficiency performance against the transmit power ranges when the *N* = 48, in Figure 6. Again, we observe the same trend as indicated in Figure 5. However, the spectral efficiency performance gain of around 2 dB is achieved by increasing the number of phase-shifting elements at the *R* RISs. Thus, by increasing the transmit power and the phase-sifting elements ∀R RISs, we achieve better spectral efficiency and the performance of the proposed solution approximates the trends of the existing solution at a very low computational cost, which shows the effectiveness of the proposed solution over the existing scheme.

**Figure 7:**
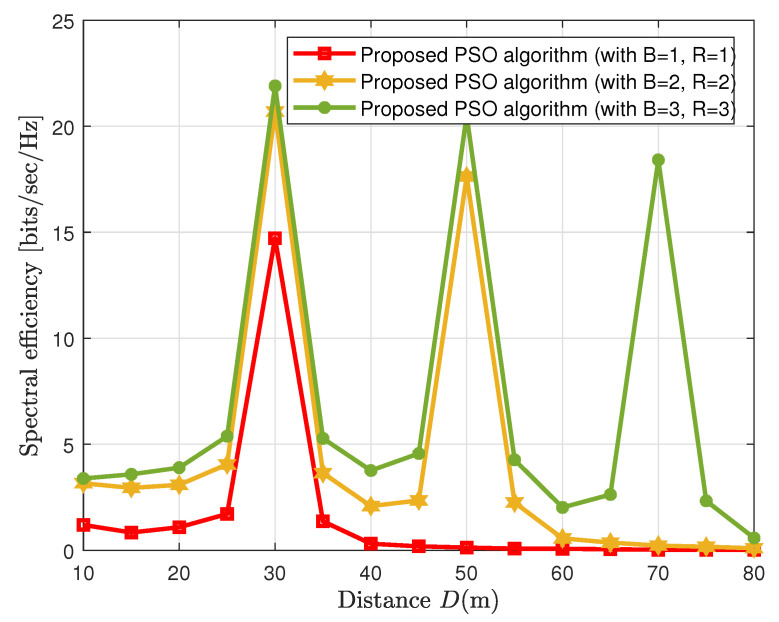
Impact of spectral efficiency on different RISs configurations.

To realize the objective of cooperative communications by aiding RISs in conventional cooperative BSs-based communications, we plot the spectral efficiency performance of the RIS-aided CF MIMO network against the distance parameter. It is clear from Figure 7 that by deploying more RISs in the cooperative scenario we can achieve higher spectral efficiency than just considering the BSs-based cooperative communications. It is observed from Figure 7 that when we install only one RIS and one BS we achieve only a single height of spectral efficiency where an RIS is installed (*D* = 30 m). However, the spectral efficiency of the cooperative RIS-aided CF network is achieved by installing more BSs and low-cost energy-efficient RISs. This not only maximizes the spectral efficiency but also minimizes the power consumption of the entire wireless system due to the presence of energy-efficient RISs.

### 4.5. Evaluation of Spectral Efficiency: Single-Antenna vs. Multi-Antenna Users

**Figure 8:**
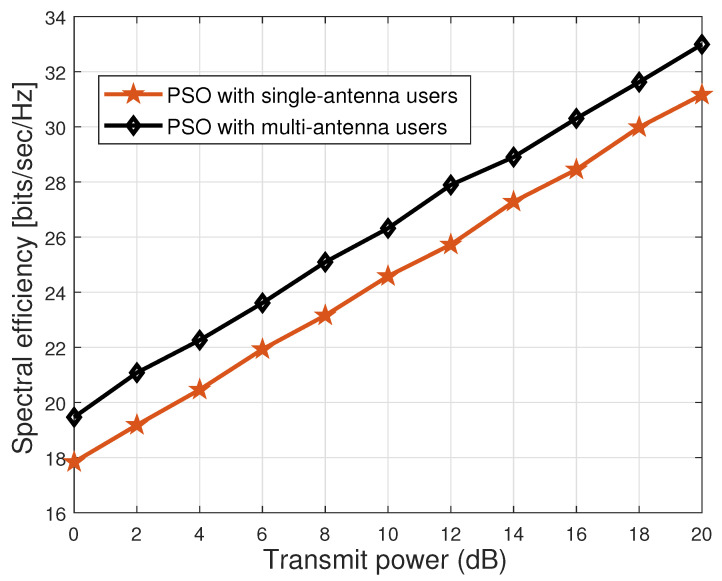
Spectral efficiency comparison between single-antenna and multi-antenna users.

We next investigate the performance of the proposed PSO scheme for both single-antenna users and multi-antenna users, as indicated in Figure 8. From Figure 8, we can see that by increasing the antennas (from 1 to 2) of the users, a performance gain of around 1.8 bits/sec/Hz is observed. This gives us an intuition that by increasing the number of antennas of the users, we can further maximize the spectral efficiency of the cooperative RIS-aided CF MIMO network.

### 4.6. Extension to the Energy Efficiency Case

We next evaluate the energy efficiency (EE) performance of the considered cooperative RIS-aided MIMO framework. To this end, based on the power consumption model discussed in [24], the overall power consumption model can be written as

(12) $$P_{tot}=\zeta {∥BG_{b}∥}^{2}+BP_{BS}+RP_{RIS}+KP_{UE}+P_{bh}$$

where $\zeta =\rho^{-1}$; $\rho^{-1}$ represents the BSs amplifier transmit power efficiency; $P_{BS}$ denotes the power consumption for the BS; $P_{RIS}$ represents the power consumption at the *r*th RIS, and finally, $P_{UE}$ and $P_{bh}$ indicate the power dissipation of the *k*th user equipment and backhaul network, respectively.

Based, on the power consumption model (12), the EE metric of the RIS-aided CF network is given by

(13) $$\begin{array}{c} EE=\frac{R_{tot}}{P_{tot}} \end{array}$$

For the EE performance metric, we consider the simulation configurations discussed in [24], the transmit power efficiency parameter ζ is set as ζ = 1.2, the $P_{BS}$ parameter for the *b*th BS is considered as 9 dBW; the power consumed by the *k*th user equipment is set to 10 dBm. Since the phase-shifting elements consume less power, as discussed in [24], we set the same parameter as in [24], i.e., $P_{RIS}$ = 10 dBm. Then, we exploit the proposed PSO algorithm to evaluate the performance of EE. This EE performance also gives us some intuition regarding the effectiveness of the proposed low-complexity PSO algorithm.

**Figure 9:**
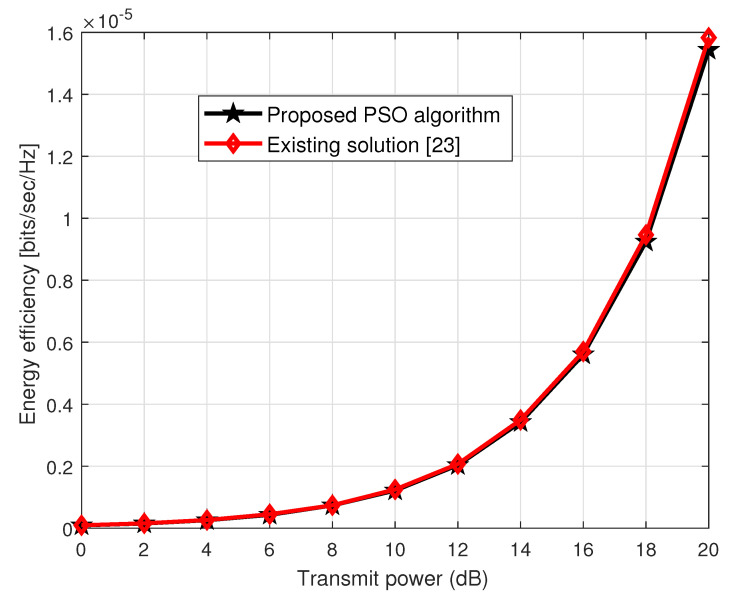
Energy efficiency performance over the wide ranges of transmit power.

Based on the EE model, we plot the EE performance against the transmit power. Since the denominator of the EE is the $P_{tot}$, which has a constant power consumption value, the major contribution of the EE will be involved in the maximization of the numerator value, which is in fact $R_{tot}$. Thus, maximizing the $R_{tot}$ will lead to maximizing the EE of the RIS-aided cooperative CF network. From Figure 9, one can see that the transmit power of the RIS-aided CF network is directly proportional to the EE. Increasing the transmit power maximizes the EE of both of the schemes, i.e., proposed and existing schemes.

## 5. Discussion

A CF network has been proposed to eliminate inter-cell interference (ICI). In the CF, a large number of BSs are installed to ensure good quality service to uniformly distributed users without cell boundaries, thus maximizing the spectral efficiency of the wireless system. However, network densification with a large number of BSs results in high hardware costs and power consumption. Nevertheless, BSs cooperative network integrated with the recently proposed energy-efficient RISs may reduce this problem and offer high spectral and energy efficiency. In this work, we aim at optimizing the beamforming solutions to maximize the spectral and energy efficiency of the RIS-aided cooperative CF MIMO network. To optimize the beamforming at the BS, we employ the ZF technique and exploit an iterative PSO solution to realize passive beamforming at the RISs. The PSO is a low-complexity suboptimal scheme, which is inspired by the social behavior of birds that fly in clusters to find their best target to feed themselves. We also present a complexity analysis to show the effectiveness of the proposed scheme over the existing scheme in the paper. The simulation results indicate that our proposed scheme achieves 94% of the spectral efficiency while approximating the same energy efficiency performance of the existing scheme.

## 6. Conclusions and Future Works

In this work, we consider a downlink RIS-aided cooperative CF network and subsequently formulate the capacity maximization problem for the considered CF network under the limitations of the constraints. Then, the proposed capacity maximization problem is solved by exploiting an iterative low-complexity PSO scheme to jointly optimize the beamforming at the BSs and the RISs, respectively. We also present the complexity analysis to indicate the strength of the proposed scheme over the existing scheme. Finally, the simulation results are provided, which indicate that our proposed scheme approximates the spectral and energy efficiency performance of the existing solution but at a very low complexity.

This paper gives us useful insight related to the low-complexity joint optimization technique to maximize the spectral efficiency of the RIS-aided CF network. However, we can extend this work to various other cases: (1) we have considered continuous phase-shifting-based beamforming at the RISs, and we can consider RISs with discrete phase-shifting elements in future works; (2) investigating the channel estimation problem when the number of RISs is included makes the problem challenging; (3) another future research direction could be the investigation of the area of RISs and how much the size of an RIS impacts the performance of the RIS-aided wireless systems. However, the larger the size of the RIS, the larger the complexity overhead. Additionally, deploying more RISs can increase the power consumption of the wireless system. Therefore, keeping these factors in mind, an optimum size of an RIS can be determined to evaluate the tradeoff between the systems’ performance and overhead complexity.

## Figures and Tables

**Table 1:** A Summary of the existing solutions with pros and cons.

Reference	Methods	Framework	Advantage	Limitations
[22]	Semidefinite relaxation (SDR)	RIS-aided MIMO system	Significantly improves spectral efficiency performance	Extremely high computational cost
[23]	Fractional programming (FP) method	RIS-aided multi-user network	Improves weighted sum rate	High computational cost
[24]	Sequential programming (SP) method	RIS-aided wireless network	Improves energy/spectral efficiency	High computational cost
[25]	Block coordinate descent (BCD) algorithm	RIS-aided multiuser network	Improves weighted sum rate	High computational cost
[26]	Penalty dual decomposition (PDD) method	RIS-aided multiuser network	Two-time scale joint beamforming scheme	High computational cost

**Table 2:** Summary of main notations.

Symbols	Meaning
**v**	Vector
**V**	Matrix
**V** ${}^{T}$	Transpose of **V**
**V** ${}^{H}$	Hermitian of **V**
**V** ${}^{†}$	Pseudo-inverse of **V**
E	Expected operator
Tr. (·)	Trace function
**V** ${}_{l1}$	*l*1 norm
**V** ${}_{l2}$	*l*2 norm
diag (·)	Diagonal entries of the matrix
*∠*	Angle of the argument

**Table 3:** A summary of simulation parameters.

Symbols and Value	Symbols and Value
*M* = 4	Noise = −120 dBm
*N* = 48	*x* = 3 for BS-user
*K* = 4	*x* = 2 for BS-RIS and RIS-user
*S* = 40	1st BS position = (0 m, −20 m)
2nd BS position = (45 m, −20 m)	1st RIS position = (30 m, −3 m)
2nd RIS position = (50 m, 3 m)	3rd RIS position = (70 m, 3 m)

## Data Availability

Not applicable.
